# Psychosocial stress induction in vivo vs. in virtuo and the influence of a health app on the acute stress reaction in youths: a study protocol for a randomized controlled trial

**DOI:** 10.1186/s13063-022-06758-z

**Published:** 2022-10-04

**Authors:** Daniel Schleicher, Angelika Ecker, Martin Kocur, Irina Jarvers, Colin Nash, Leonie Götz, Alexandra H. Otto, Stephanie Kandsperger, Romuald Brunner

**Affiliations:** 1 Clinic of Child and Adolescent Psychiatry, Psychosomatics and Psychotherapy, University of Regensburg, Universitätsstraße 84, 93053 Regensburg, Germany; 2 Department of Media Informatics, University of Regensburg, Universitätsstraße 31, 93053 Regensburg, Germany

**Keywords:** Trier Social Stress Test, Virtual reality, Ambulatory Assessment, Cortisol, Stress mechanisms, Coping, Children and adolescents, Relaxation, Eye-tracking

## Abstract

**Background:**

Acute and everyday stress is substantial for the development of mental and physical diseases, therefore it is crucial to get a better understanding of its pathogenesis. Different methods (e.g., Ambulatory Assessment) and stress reactivity paradigms (e.g., Trier Social Stress Test / TSST) in laboratory settings are often used to investigate basic mechanisms of this process. Due to the technological progress of the last years and especially due to children and adolescents growing up with it, the application of these developments in clinical research is reasonable. The aim of this project is to successfully transfer the TSST for children and adolescents into the virtual world, which will be compared to a real TSST situation. Physiological and psychological stress reactions will be analyzed in order to assess similarities and differences. Moreover, it will be investigated whether a Heart Coherence Exercise (HCE) has a stronger influence on coping with acute stress compared to Natural Relaxation (NR).

**Methods:**

This single-center experimental study will examine acute and everyday stress and coping processes in eighty-four healthy children and adolescents between the ages of 11 and 17. For everyday stress, different parameters (e.g., hormonal profiles and mood ratings) as well as a history of stressful life events and utilized coping methods will be recorded and a relaxation exercise will be practiced on a smartphone over 2 days. Regarding the acute stress reaction, the participants will be confronted either with the virtual or the real version of the TSST, followed by the trained relaxation exercise (HCE vs. NR). Physiological (e.g., cortisol and heart rate) and psychological stress markers (e.g., mood and gaze behavior) will be recorded continuously.

**Discussion:**

Studies are sparse using a virtual version of the TSST in children and adolescents. A successful virtual TSST would constitute an economical variant, which would also make it easier to administer it in clinical or population-based samples. Effective ambulatory relaxation exercises would be a useful addition to clinical treatment approaches.

**Trial registration:**

The study is registered in the German Clinical Trials Register since 10 August 2020 (DRKS00022063).

## Administrative information

Note: the numbers in curly brackets in this protocol refer to SPIRIT checklist item numbers. The order of the items has been modified to group similar items (see http://www.equator-network.org/reporting-guidelines/spirit-2013-statement-defining-standard-protocol-items-for-clinical-trials/).

**Table:** 

Title {1}	Psychosocial stress induction in vivo vs. in virtuo and the influence of a health app on the acute stress reaction in youths: a study protocol for a randomized controlled trial
Trial registration {2a and 2b}.	Registration in the German Clinical Trials Register since 10 August 2020 (DRKS00022063) https://www.drks.de/drks_web/navigate.do?navigationId=trial.HTML&TRIAL_ID=DRKS00022063
Protocol version {3}	05.09.2022, Version 3.0
Funding {4}	This study will be financed by the Clinic of Child and Adolescent Psychiatry, Psychosomatics and Psychotherapy, University of Regensburg, under the direction of Prof. Dr. med. Romuald Brunner. The design of the study, the collection, analysis and interpretation of data as well as the writing of the manuscript will not be funded externally.
Author details {5a}	Daniel Schleicher, M. Sc., M. A. * Daniel.Schleicher@medbo.de Clinic of Child and Adolescent Psychiatry, Psychosomatics and Psychotherapy, University of Regensburg, Universitätsstraße 84, 93053 Regensburg, Germany Angelika Ecker, M. Sc. * Angelika.Ecker@ukr.de Clinic of Child and Adolescent Psychiatry, Psychosomatics and Psychotherapy, University of Regensburg, Universitätsstraße 84, 93053 Regensburg, Germany * Daniel Schleicher and Angelika Ecker contributed equally to this work and share first authorship. Martin Kocur, M. Sc. Martin.Kocur@ur.de Department of Media Informatics, University of Regensburg, Universitätsstraße 31, 93053 Regensburg, Germany Irina Jarvers, M. Sc. Irina.Jarvers@ukr.de Clinic of Child and Adolescent Psychiatry, Psychosomatics and Psychotherapy, University of Regensburg, Universitätsstraße 84, 93053 Regensburg, Germany Colin Nash Colin.Nash@stud.uni-regensburg.de Clinic of Child and Adolescent Psychiatry, Psychosomatics and Psychotherapy, University of Regensburg, Universitätsstraße 84, 93053 Regensburg, Germany Leonie Götz Leonie.Goetz@medbo.de Clinic of Child and Adolescent Psychiatry, Psychosomatics and Psychotherapy, University of Regensburg, Universitätsstraße 84, 93053 Regensburg, Germany Alexandra H. Otto, M. Sc. Alexandra.Otto@ukr.de Clinic of Child and Adolescent Psychiatry, Psychosomatics and Psychotherapy, University of Regensburg, Universitätsstraße 84, 93053 Regensburg, Germany Dr. med. Stephanie Kandsperger Stephanie.Kandsperger@medbo.de Clinic of Child and Adolescent Psychiatry, Psychosomatics and Psychotherapy, University of Regensburg, Universitätsstraße 84, 93053 Regensburg, Germany Prof. Dr. med. Romuald Brunner Romuald.Brunner@medbo.de Clinic of Child and Adolescent Psychiatry, Psychosomatics and Psychotherapy, University of Regensburg, Universitätsstraße 84, 93053 Regensburg, Germany
Name and contact information for the trial sponsor {5b}	Prof. Dr. med. Romuald Brunner Romuald.Brunner@medbo.de Clinic of Child and Adolescent Psychiatry, Psychosomatics and Psychotherapy, University of Regensburg, Universitätsstraße 84, 93053 Regensburg, Germany
Role of sponsor {5c}	This study will be financed by the Clinic of Child and Adolescent Psychiatry, Psychosomatics and Psychotherapy, University of Regensburg, under the direction of Prof. Dr. med. Romuald Brunner. The design of the study, the collection, analyses and interpretation of data as well as the writing of the manuscript will not be funded externally.

## Introduction

### Background and rationale {6a}

Stressful or traumatic experiences during childhood are among the most important risk factors for the development of a broad range of mental and physical diseases in child and adulthood [1–3]. Strong relationships have been found between the extent of stress experiences in childhood and the occurrence of various disorders in adulthood. These include depression, anxiety disorders, addictions, and personality disorders, but also physical conditions such as cardiovascular diseases, respiratory diseases, obesity, diabetes, pain disorders, and autoimmune diseases [4].

A definable stress reaction manifests itself in different domains: physiological (heart rate, skin conductance, hormone release), psychological (emotional, cognitive, interpretative), and behavioral (attention, arousal, alertness) [5]. In laboratory settings, these markers can be investigated under controlled conditions, e.g., using the Trier Social Stress Test (TSST) where acute stress is triggered effectively [6], both in adults [7] and children [8]. The main components of the TSST are an interview before a non-confirming, neutral panel, and a spontaneous arithmetic task. Within this paradigm, the factors of social evaluation, uncontrollability, and unpredictability trigger a stress response at different levels [7]. For the TSST, there are also already successfully evaluated variations, e.g., for younger [9] or older [10] participants as well as virtual versions [11, 12] for adults. A virtual version of the TSST for younger participants has neither been investigated nor validated and only one successful application has been published for adolescents with excess weight so far [13]. To the knowledge of the authors, an evaluation of the transferability and applicability of a virtual version to children or adolescents has not yet been carried out. The advantages of a virtual version are a good access format for the younger generation, far-reaching context control and reduced resource requirements in the application of the TSST [12]. These benefits may also help to simplify and conduct more accessible research on acute stress reactions and coping mechanisms in children and adolescents in general.

Beyond acute and clearly defined stressful situations, daily stressors should also be considered in the investigation of stress in adolescence and childhood. To minimize retrospective biases, a physiological Ambulatory Assessment (AA) can be used. Several measurements, both hormonal and subjective parameters, can thus be collected in the natural environment of the participant. Of particular interest are, besides the Cortisol Awakening Response (CAR), diurnal profiles of cortisol or alpha-amylase, for example [14].

The counterpart to acute and everyday stress is coping. The extent of the stress experience depends among others on the individual’s ability to cope with stress. There are many possible approaches investigating coping mechanisms in children and adolescents, the positive effects of such stress-reducing methods have already been observed in the acute stress reaction triggered by the TSST. In one study of Domes and colleagues [15], different methods (Internet-Based Stress Management vs. Progressive Muscle Relaxation) trained over several weeks showed a reduction in subjective stress perception. Internet-Based Stress Management even showed significantly lower cortisol release compared to the control group [15]. Very simple and therefore well suited for children and adolescents and simultaneously easily accessible methods are breathing exercises. Despite their simplicity, they often require not only instruction but also regular training until a stressed person can use them well [16]. A variation that does not require much training is a Heart Coherence Exercise (HCE). During this exercise, 5 s of inhalation and 5 s of exhalation are taken over 5 min. It could be shown that such an HCE can improve cognitive abilities and hyperactive behavior among other things [17]. New digital media such as smartphone health apps offer a wide range of choices for stress regulation (e.g., meditation, mood and sleep diaries), even if they are hardly scientifically based [18]. The increased accessibility of technology, especially for the young generation, provides a low-threshold offer for stress regulation which can be of important value. The HCE is already available as a smartphone app and integrated as a breathing exercise in many smartwatches.

### Objectives {7}

The project focuses on the successful transferability of the TSST for children and adolescents from reality to virtual reality (VR) and the effectiveness of a breathing exercise immediately after an acute stress induction. For this purpose, physiological, psychological, and behavioral variables will be collected in order to analyze and compare the increases, decreases, and changes of acute stress reactions in both TSST versions. To examine everyday stress reactions in healthy children and adolescents, the changes in different physiological and psychological stress parameters will also be investigated for 2 days at home.

It is hypothesized that there will be significant increases in physiological stress parameters (cortisol, alpha-amylase and heart rate) and significant changes in psychological stress responses (mood ratings and gaze behavior) to the TSST in vivo and in virtuo. Specifically, it is expected that both the real and virtual stress situation will cause similar stress responses in these variables. Moreover, it is expected that the HCE will cause a significantly greater decrease in these stress parameters compared to Natural Relaxation (NR). Significant associations between changes of everyday psychological (subjective sleep quality, mood ratings, stressful events, and coping skills) and physiological variables (awakening responses and diurnal profiles of cortisol and alpha-amylase) are expected.

### Trial design {8}

**Fig. 1:**
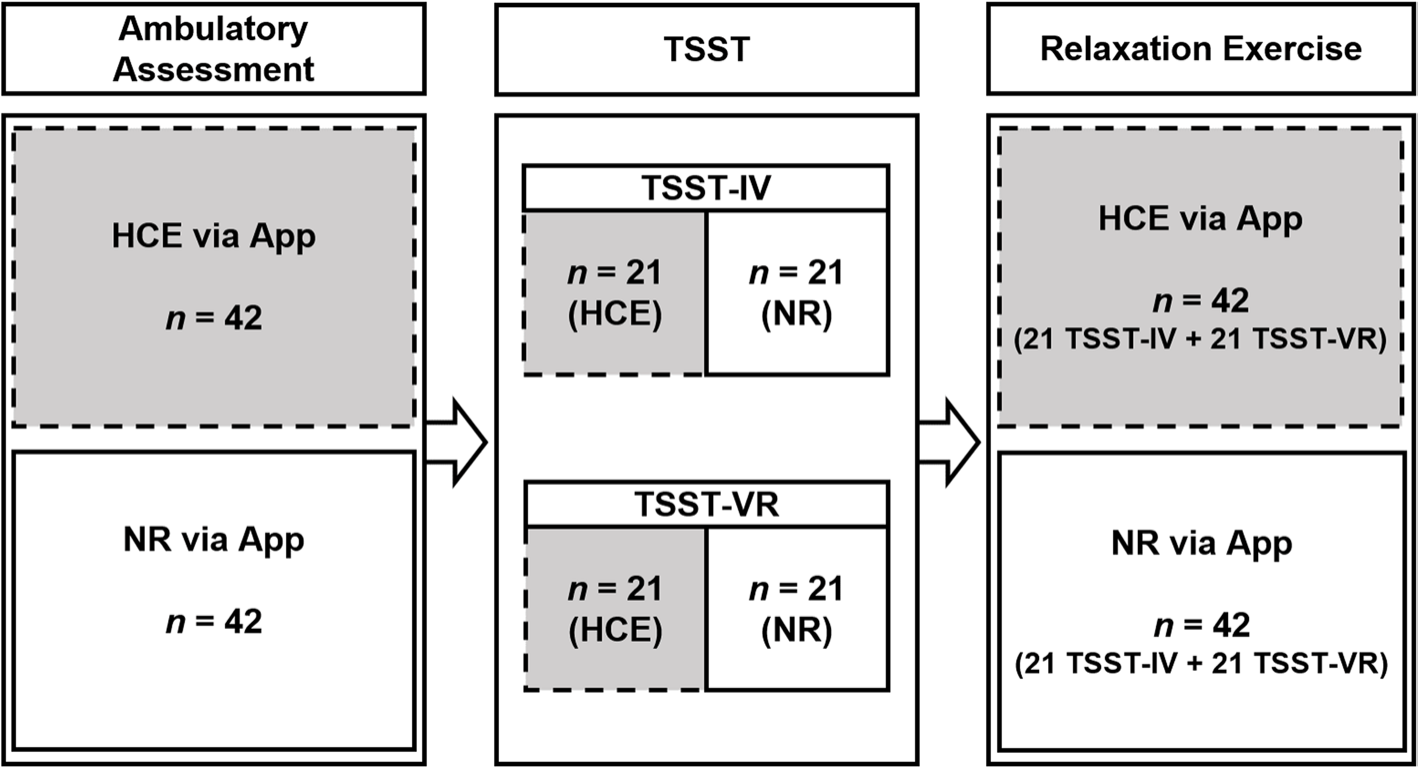
Overview of study design. HCE, Heart Coherence Exercise; NR, Natural Relaxation; TSST, Trier Social Stress Test; TSST-IV, Trier Social Stress Test in vivo; TSST-VR, Trier Social Stress Test in virtual reality

This study is planned as a 2×2 design (Fig. 1), with the independent variables “relaxation exercise after acute stress” (Heart Coherence Exercise / HCE vs. Natural Relaxation / NR) and “stress induction by TSST” (TSST in virtuo / TSST-VR vs. TSST in vivo / TSST-IV). The assignment to the resulting 4 groups will be randomized (allocation ratio of 1:4). In the relaxation exercise after acute stress, a superiority of HTC over NR is expected; in stress induction by TSST, an equivalence between TSST-IV and TSST-VR is expected.

## Methods: participants, interventions and outcomes

### Study setting {9}

Children and adolescents will be interviewed and tested by psychologists in the research facilities of the Clinic of Child and Adolescent Psychiatry, Psychosomatics and Psychotherapy of the University of Regensburg, Germany. In addition, several surveys will be conducted at home over 2 days using a smartphone app.

### Eligibility criteria {10}

A prerequisite for participation in the planned experiment will be an age between 11 and 17 years. Another inclusion criterion will be sufficient understanding of the German language. Children and adolescents who are or have ever been undergoing psychiatric, psychotherapeutic, or neurological treatment will be excluded as well as participants with current psychiatric disorders. This will be ruled out by conducting a structured clinical interview. Children and adolescents who are taking glucocorticoid-containing medication and / or psychotropic drugs will also be excluded. In addition, children and adolescents with neurological, endocrinological, or immunological (pre-)diseases will be excluded. Further exclusion criteria will be pregnancy / lactation, early pubertal development due to a hormone status deviating from the norm, intellectual impairment (IQ < 85), and attendance in a school for special needs. Before children and adolescents will be invited to participate in the study, a short screening on the phone will be carried out. Here, inclusion and exclusion criteria will be asked for in order to determine a possible exclusion in advance and to minimize the effort for the children and adolescents.

### Who will take informed consent? {26a}

At the first appointment (T1), a detailed explanation of the study will be provided to the children / adolescents and their parents / legal guardians. This will be done orally and a written information will be handed out additionally. Any questions and uncertainties that arise can be clarified immediately with a person from the study personnel. Consent forms will be signed by the participant, the accompanying parent / legal guardian, and a person from the study personnel. All participants will agree upon taking part voluntarily and termination of their participation is possible at any time without giving any reasons.

### Additional consent provisions for collection and use of participant data and biological specimens {26b}

The collected data will serve only scientific purposes. It will be collected and stored pseudonymously. The saliva samples will be numbered consecutively and analyzed in an external laboratory (Dresden LabService GmbH, 01307 Dresden, under the leadership of Prof. Dr. Clemens Kirschbaum, Professorship Biopsychology, Technical University of Dresden, Germany). The data of the health app will be stored temporarily on research smartphones of the department until the data is transferred manually by a member of the study personnel. After the data is saved on an extern hard drive, it will be deleted from the smartphone. The coding key for the pseudonyms will be destroyed 1 year after completion of the study, which will make the participant data anonymous. Study data will only be published in anonymous form.

## Interventions

### Explanation for the choice of comparators {6b}

The comparison between TSST-IV and TSST-VR was chosen in order to evaluate the transferability of the TSST into a virtual version, which would provide various benefits (e.g., lower costs and control of context variables). The comparison between HCE and NR serves to evaluate the effectiveness of the guided relaxation exercise after acute stress compared to a natural control condition.

### Intervention description {11a}

After a telephone screening (T0) for checking the inclusion and exclusion criteria, the participants and their parents / legal guardians will be invited to an initial appointment (T1) in our research facilities. Female subjects who are already menstruating will only be tested (AA and T2) in the luteal phase to ensure comparability with the cortisol reactivities to the male subjects [19].

At the first appointment (T1), a detailed briefing for the participants and the parents / legal guardians will be carried out by an experimenter. After answering all upcoming questions and signing the informed consent, an intelligence test and a clinical interview with the participants will be conducted to check the exclusion criteria. Furthermore, the participants will complete questionnaires on puberty status, personality, traumatic experiences, temperament, and behavior. In preparation for the AA, the participants will be instructed in how to use the salivettes (Sarstedt, Nümbrecht, Germany) for collecting saliva samples. Moreover, the usage of the health app including short surveys and the relaxation exercise (developed and programmed in cooperation with the Department of Media Informatics, University of Regensburg) will be explained and demonstrated to the participants. The app was implemented with the integrated development environment Android Studio. The code was written in Kotlin and Java for the target SDK version 29. To develop the questionnaires, the programming library SurveyKit, Version 1.1.0 [20] was used, which is released under the open source MIT License. The application will run on a Samsung A20e with the operating system Android Pie 9.0 installed. Any questions the participant may have regarding the use of the app can be clarified with the experimenter at this point. The first appointment will take approximately 2.5 h.

**Fig. 2:**
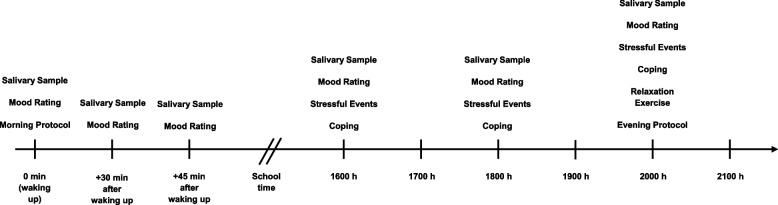
Timeline of Ambulatory Assessment

The AA will take place on two consecutive weekdays. The first saliva sample will be collected simultaneously with the first alarm clock. The participants will receive a notification via the smartphone app to use the first salivette. A timer will be activated and 30 min later the smartphone app will send another notification to use the second salivette. Between the first and second salivette, the participants will receive information about a 20-min time window for breakfast, because they should not eat anything 10 min before the next salivette to avoid contamination. Subsequently, another timer of 15 min will start. Then, the third and last saliva sample for the registration of the alpha-amylase and cortisol awakening responses will be collected. While the salivette should be kept inside the mouth for about at least 2 min, the participants will be asked about their sleep (only during the first salivette) and their mood (during all three salivettes) via the smartphone app. Overall, the AA in the morning will take place within a time frame of about 45 min. During school time (0800–1600 h), no notifications will appear on the smartphone. At 1600, 1800, and 2000 h, a saliva sample will be taken and stressful events since the last survey as well as used coping strategies and the current mood will be queried. In addition, a relaxation exercise will be carried out for 5 min during the survey at 2000 h. One half of the participants will be given the task of performing the HCE (breathing in and out for 5 s each) guided by the app, while the other half will be given the task of remaining calm and relaxing naturally during this time. At the end of the session, the participants will be asked to assess their mood and to give an overall assessment of the day and if there were any technical problems with the smartphone app. This procedure will be repeated the same way on the second day of the AA (Fig. 2).

**Fig. 3:**
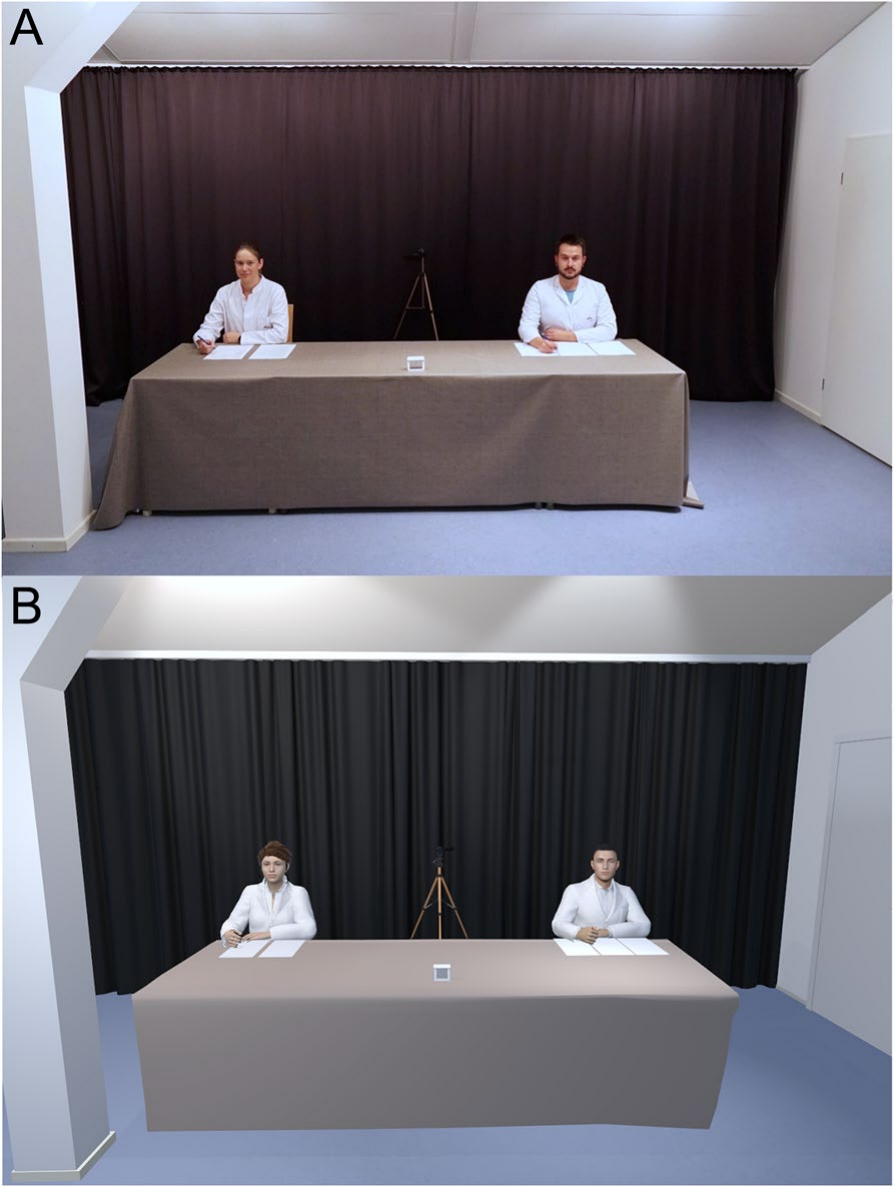
Comparative presentation of the Trier Social Stress Test in vivo (**A**) and in virtual reality (**B**)

The second test appointment (T2) will again take place in our facilities and the TSST will be carried out with the participants. Due to the hormonal circadian rhythm and to record greater cortisol responses, all participants will be tested in the afternoon [21]. Furthermore, 8 saliva samples and 9 mood assessments will be taken to capture the psychological and endocrinological stress reactions. Directly after arrival of the participants, the first saliva sample will be taken and the sensor for measuring the heart rate will be attached (about 20 min after arrival). The participants can come to rest by filling out questionnaires and using relaxation material. This will be followed by the TSST, whereby VR glasses or eye-tracking glasses will be first put on, calibrated, and tested before the preparation period of the TSST (5 min) starts. Then, the participants will be confronted with a speech (“Why are you suited for the position of student representative?”) and an arithmetic task (e.g., for 11-year-old participants: “Please count backwards from the number 785 in steps of 7.”) in front of a panel and a camera. Both tasks will be adapted to the age of the participants and last 5 min each. In the TSST-IV, the panel will consist of one female and one male staff member of the department, who have been specially trained to keep gestures, facial expressions, and feedback neutral. Based on the real version, we will use one male and one female virtual human in the TSST-VR. We designed the virtual humans using the computer graphics software Daz3D [22]. We used the characters Genesis 8 Male and Genesis 8 Female [23] and adapted their appearance (body shape, facial expressions, skin, lip synchronization) by morph targets and textures to match the skin type and basic appearance to the real panel members in the TSST-IV. The virtual humans can give visual (taking notes) and auditory (e.g., “Error, please start again.”) feedback that can be triggered by the experimenter via keyboard. These feedback options are similar to the reactions of the panel in the real version. We implemented the VR application using the Unity3D game engine (v. 2019.3.11f1) and modelled a virtual replica of the real environment with identical room dimensions (width: 5.82 m, length: 5.85 m) using the computer graphics software Blender [24] (Fig. 3). We included 3D models of all objects placed in the real environment to ensure the virtual space consists of the same accessory with the same number of potential distractors. To register fixations of the gaze behavior, we defined five 3D Areas of Interest (AOI) using invisible bounding boxes around the male and female virtual human’s face and body, and the camera. We used the maximum bounding box of each AOI to define the *x*, *y*, and *z* dimensions [25]. To immerse the participants in the virtual environment, they will be fitted with an HTC Vive Pro Eye Head Mounted Display (HMD). The HMD has a wide horizontal field of view of 110° and a spatial resolution of 1440 × 1600 pixels per eye. All audio files will be presented via the HMD’s headphones. Participants in VR will perceive the surrounding environment from first-person perspective. The VR application will run on a Dell G5 15 notebook PC (Windows 10, Intel i7-8750H, 8GB RAM, NVIDIA GeForce RTX 2060 Mobile graphics card).

**Fig. 4:**
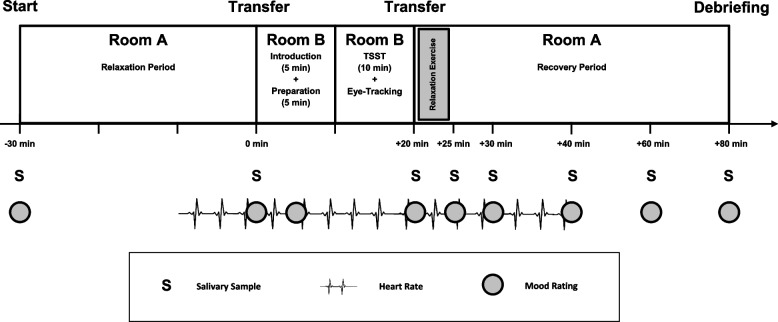
Timeline of the second test date. S, Salivary Sample; TSST, Trier Social Stress Test

The real or virtual TSST will immediately be followed by the 5-min relaxation phase via the smartphone app. The participants shall relax as practiced in the AA. Therefore, in the control group they should sit calmly and relaxed and in the intervention group they should relax by breathing with the help of the smartphone app. Afterwards, they shall use the remaining time to answer further questionnaires at rest. During the debriefing, particular attention will be paid to conveying to the participants that the committee of the TSST was only trained personnel of the research team or programmed avatars. They were supposed to behave in this way regardless of the performance of the participant. It will be made clear that it was not a measurement of individual performance but of psychosocial stress. In total, this appointment will take approximately 2 h. An overview of the procedure for the second test date is shown in Fig. 4.

### Criteria for discontinuing or modifying allocated interventions {11b}

If the diagnostic instruments used to check the inclusion criteria show abnormalities during the telephone screening (T0) or at the first appointment (T1) and exclusion criteria are thus met, study participation will be discontinued at this point and the person will be excluded from further study participation.

During the telephone screening (T0), participants will be asked in advance if they have ever suffered from severe motion sickness (e.g., “Have you ever been sick on a ship or while reading a book in a car?”) as an indicator for VR sickness. If so, the participants will be excluded from the TSST-VR condition and assigned to the TSST-IV group. Regarding the change between TSST conditions, there are no other reasons besides severe motion sickness that will lead to a change between groups.

### Strategies to improve adherence to interventions {11c}

For each survey in the AA, there will be a narrowly defined time period during which participants can answer questions. The participants will receive a notification on the research smartphone. If there is no reaction to the notification, the app will send additional reminders (about every 3 min). When a notification is accepted and answered, the participants will receive a message that the survey was successful.

Furthermore, the participants will be given containers with Medication Event Monitoring System (MEMS) Caps (AARDEX Group, Liège, Belgium) for the transport and storage of the salivettes. These MEMS Caps are used to improve the adherence to the collection of saliva samples. By opening the containers, the time is registered and controlled. This is also communicated to the participants in advance.

After completing both appointments (T1 and T2), children and adolescents will receive a gift voucher worth 40 euros for their participation. In addition, they will receive another 10 euros if they complete at least 70% in the AA.

### Relevant concomitant care permitted or prohibited during the trial {11d}

Psychiatric, psychotherapeutic, and neurological treatment as well as the use of corticoid-containing and/or psychotropic drugs will not be allowed during study participation, as these correspond to exclusion criteria of our study. Otherwise, there will be no restrictions on concomitant patient care during the trial.

### Provisions for post-trial care {30}

During the test appointment (T2), the participants will be confronted with a stress paradigm in a virtual or real environment, which represents a moderate psychosocial stress induction. We do not expect excessive and long-lasting emotional distress for the participants, since the paradigm resembles everyday school and performance situations and is often used in psychosocial stress research. Furthermore, the participants will be healthy children and adolescents and the participation will be voluntary. If adverse effects (e.g., high stress levels or VR sickness) during the stress induction at T2 occur, they will be noted in detail on a protocol. These adverse effects are, if present, only short term. In this case, the study personnel as psychological experts will always be on site as contact persons. In summary, because we do not expect long-lasting adverse effects beyond the study date, no post-trial care is necessary.

### Outcomes {12}

#### Primary outcome measures

The primary outcome is the comparison of changes in stress responses between the two TSST conditions and relaxation groups. Acute stress reactions will be measured on psychological and physiological level. Self-reported assessments of acute stress / tension, sadness, anxiety / fear, anger, and aggression will be recorded. Moreover, heart rate, cortisol, alpha-amylase, and gaze behavior will be analyzed to compare the changes of these stress responses between the TSST paradigms and the relaxation exercises. In summary, there are several primary outcome variables: mood, cortisol, alpha-amylase, heart rate, and gaze behavior. The reactions will be recorded multimodally to be able to analyze different levels of stress regulation comprehensively.

#### Secondary outcome measures

The second outcome is the change in stress responses in everyday life. Everyday stress reactions will be recorded in the AA via self-report and endocrinological measures at a total of 12 time points. Important variables will be subjective evaluations of sleep quality and quantity, stress / tension, sadness, anxiety / fear, anger, and aggression. Furthermore, the participants will be asked to report stressful events of the day and which coping skills they have used. Cortisol and alpha-amylase will be assessed to analyze endocrinological awakening responses and diurnal hormonal profiles. In summary, there are several secondary outcome variables: mood, cortisol, alpha-amylase, stressful events, coping skills, sleep quality, and quantity.

#### Other outcomes of interest

In addition, personality and temperament traits, intelligence, impulsiveness, aggression, anxiety, trauma, and other psychological variables will be recorded with the help of various psychometric questionnaires and interviews. The variables will be used to characterize the sample and for exploratory correlation analyses, for example, between personality traits and stress responses.

### Participant timeline {13}

**Fig. 5:**
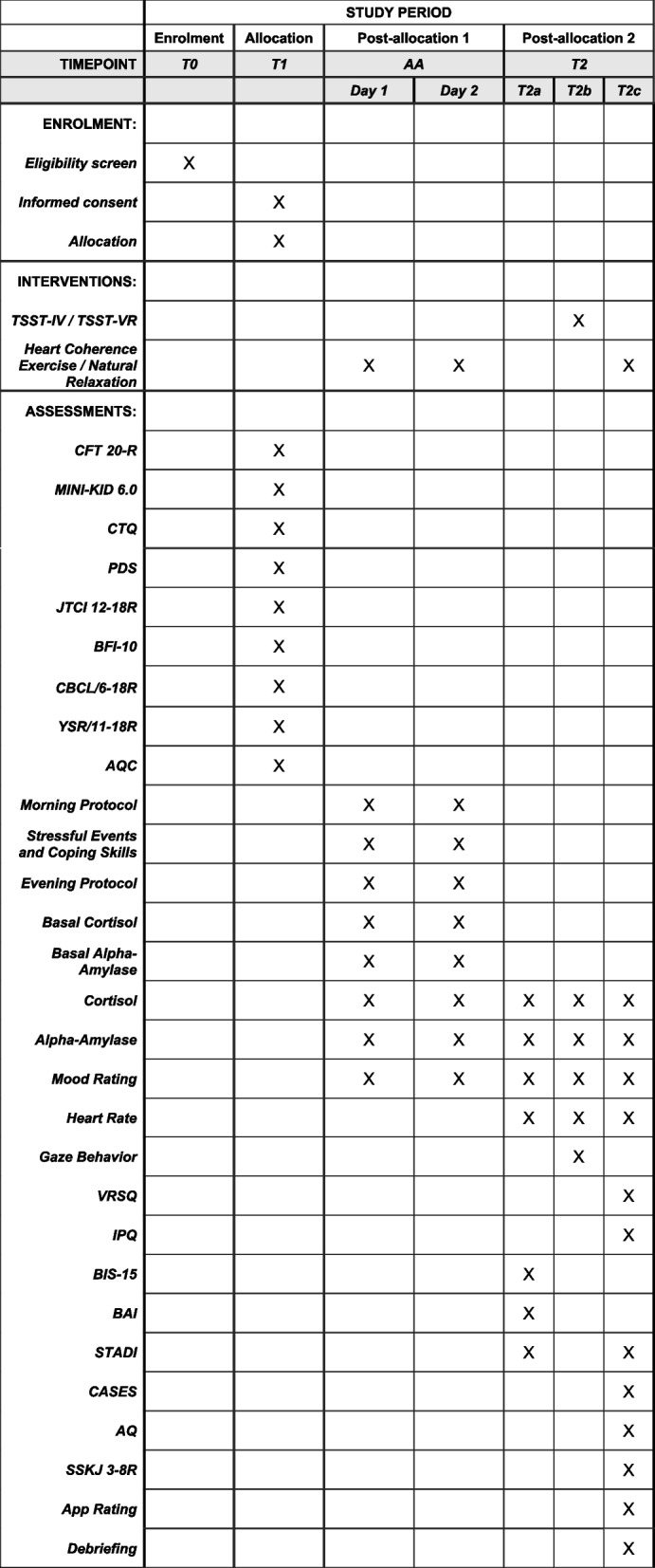
Time schedule of enrolment, interventions and assessments. TSST, Trier Social Stress Test; TSST-IV, Trier Social Stress Test in vivo; TSST-VR, Trier Social Stress Test in virtual reality; CFT 20-R, Culture Fair Intelligence Test 20-R – Scale 2; MINI-KID 6.0, Mini-International Neuropsychiatric Interview for Children and Adolescents 6.0; CTQ, Childhood Trauma Questionnaire; PDS, Pubertal Development Scale; JTCI 12-18 R, Junior Temperament and Character Inventory 12-18 R; BFI-10, 10 Item Big Five Inventory; CBCL/6-18R, Child Behavior Checklist 6-18 R (answered by parents and legal guardians); YSR/11-18R, Youth Self-Report 11-18 R; AQC, Alexithymia Questionnaire for Children; VRSQ, Virtual Reality Sickness Questionnaire; IPQ, IGroup Presence Questionnaire; BIS-15, Barratt Impulsiveness Scale – Short Version; BAI, Beck Anxiety Inventory; STADI, State-Trait-Anxiety-Depression-Inventory; CASES, Cognitive, Affective and Somatic Empathy Scales; AQ, Aggression Questionnaire; SSKJ 3-8 R, Questionnaire for the Measurement of Stress and Coping in Children and Adolescents 3-8 R; T0, telephone screening; T1, first test date; AA, Ambulatory Assessment; T2, second test date; T2a, pre-TSST; T2b, TSST; T2c, post-TSST

In this study, various interventions, questionnaires, and assessments will follow a fixed participant timeline (Fig. 5).

### Sample size {14}

To estimate the needed sample size, an a priori power calculation was carried out using G*Power [26]. Based on the medium average effect size of a recent meta-analysis of Helminen, Morton, Wang, and Felver [7], an effect size of *f* = .30 was expected. Therefore, a sample size of 84 persons was determined to achieve a power of 95%. Thus, the currently planned study aims at a sample size of *N* = 84. The allocation of participants to the groups will be randomized.

### Recruitment {15}

The healthy participants will be recruited from the Upper Palatinate region in Bavaria, Germany. The recruitment will be done via (online) study flyers, email distribution lists, and advertisements in educational institutions.

## Assignment of interventions: allocation

### Sequence generation {16a}

The participants will be assigned to one of the 4 groups by drawing from a uniform distribution via a pseudo random number generator. A limiting parameter during the assignment process for randomization will be the tolerability of VR. If participants indicate in the screening that they are very susceptible to dizziness (motion sickness), this will be a precautionary criterion for exclusion from the TSST-VR condition.

### Concealment mechanism {16b}

Allocation will be made after the telephone screening. Participants receive a consecutive number which is assigned randomized to one of the 4 groups by the computer software.

### Implementation {16c}

Participants will be registered after they have voluntarily contacted the study management and the inclusion and exclusion criteria have been checked. After completion of T0, study personnel will perform group assignment. For this purpose, the study personnel enters the group number of 4 groups into the pseudo random number generator, which then generates the allocation sequence and assigns the participants to the conditions.

## Assignment of interventions: blinding

### Who will be blinded {17a}

Participants will be blinded regarding the objective of the TSST and the relaxation exercise variations. At no time, participants will be aware that there are two relaxation exercises and that they may be in the control group. Participants will know from the recruitment that there is a virtual and a natural setting but are not aware of the composition of the setting.

### Procedure for unblinding if needed {17b}

There are no expected circumstances for unblinding participants during the study.

## Data collection and management

### Plans for assessment and collection of outcomes {18a}

During the short telephone screening, questions will be asked about schooling, employment, illnesses, and medication intake in order to check the inclusion and exclusion criteria.

To measure stress levels, subjective mood will be registered using 10-item Likert scales that cover the emotional components stress / tension, sadness, anxiety / fear, anger, and aggression (e.g., “At the moment I feel stressed, tensed or charged.”). For the psychophysiological measurement of hormone concentrations, heart rate and gaze behavior, salivettes, electrocardiogram / activity sensors (EcgMove 4 by movisens), and eye-tracking systems (Tobii Pro Glasses 2 and Tobii Eye-Tracking built in HTC VIVE Pro Eye Virtual Reality Glasses) will be used.

During the AA, subjective mood ratings and hormone concentrations of cortisol and alpha-amylase will be measured as mentioned above. Subjective sleep quality / quantity and the evaluation of the day will be assessed by means of morning and evening protocols (e.g., “How good and restful was your sleep?” or “How stressful was today overall?”) [27]. The nature and intensity of stressful events in the domains of family, friends, school, and health since the last survey will be registered at several time points followed by questions on used coping strategies (e.g., “I have recovered.” or “I’ve enlisted help from others.”) [28, 29].

The first test part of the Culture Fair Intelligence Test 20-R – Scale 2 (CFT 20-R) [30] will be used to estimate participants’ intelligence. In general, the test consists of two parts (part 1: 56 items, part 2: 45 items) with four sub-tests each (classification, series, analogies, and matrices).

To assess possible behavioral and mental problems, the Mini-International Neuropsychiatric Interview for Children and Adolescents 6.0 (MINI-KID 6.0) [31], the Child Behavior Checklist 6-18 R (CBCL/6-18R) [32], and the Youth Self-Report 11-18 R (YSR/11-18R) [32] will be used. The MINI-KID 6.0 is a structured clinical interview for the assessment of psychiatric disorders with the help of diagnostic sections (A–X) and dichotomous response format (Yes-No). The CBCL/6-18R (answered by parents and legal guardians) and the YSR/11-18R (answered by children and adolescents) enable the analysis of 8 scales (Anxious / Depressed, Withdrawn / Depressed, Aggressive Behavior, Rule-Breaking Behavior, Somatic Complaints, Attention Problems, Social Problems and Thought Problems) and 3 total scores (Total Problems, Internalizing Broad Band and Externalizing Broad Band Score).

The Pubertal Development Scale (PDS) [33] will be collected to assess the puberty status of the participants.

The assessment of temperament (Novelty Seeking, Harm Avoidance, Reward Dependence and Persistence) and character (Self-directedness, Cooperativeness and Self-transcendence) will be done via the Junior Temperament and Character Inventory 12-18 R (JTCI 12-18 R) [34]. The five personality traits Neuroticism, Extraversion, Openness to Experience, Conscientiousness, and Agreeableness will be measured by the 10 items of the 10 Item Big Five Inventory (BFI-10) [35].

The intensity of anxiety and depression will be assessed by the 21 items of the Beck Anxiety Inventory (BAI) [36, 37] and the state and trait scales (20 items each) of the State-Trait-Anxiety-Depression-Inventory (STADI) [38]. Moreover, aggression will be measured by the 29 items, summarized in 4 scales (Anger, Hostility, Physical and Verbal Aggression) of the Aggression Questionnaire (AQ) [39, 40]. The 15 items and 3 scales (Non-planning, Motor and Attentional Impulsivity) of the Barratt Impulsiveness Scale – Short Version (BIS-15) [41, 42] will be used to assess the nature and intensity of impulsive behavior. In addition, to measure 3 empathy domains (Cognitive, Affective, and Somatic) with 2 valence facets (Positive and Negative), the Cognitive, Affective and Somatic Empathy Scales (CASES) [43, 44] are used, which consist of 30 items. Alexithymia as a cluster of emotional processing deficits will also be assessed using the 20 items and 3 dimensions (Difficulties Identifying Emotions, Difficulties Describing Emotions and Externally Oriented Thinking) of the Alexithymia Questionnaire for Children (AQC) [45, 46].

The survey of various traumas in the past (sexual, emotional and physical abuse, emotional, and physical neglect) will be done via the Childhood Trauma Questionnaire (CTQ) [47, 48] consisting of 28 items.

Coping of everyday stressful events will be assessed by the subscales (Problem Solving, Social Support, Avoidant Coping, Palliative Emotion Regulation and Anger-related Emotion Regulation) of the Questionnaire for the Measurement of Stress and Coping in Children and Adolescents 3-8 R (SSKJ 3-8 R) [49].

In the TSST-VR condition, the feeling and experience of being in a virtual environment will be measured by the IGroup Presence Questionnaire (IPQ) [50] consisting of 14 items. Unpleasant physical sensations caused by VR will be registered by the Virtual Reality Sickness Questionnaire (VRSQ) [51] consisting of 9 items.

The questionnaires are sufficiently reliable and therefore constitute standard measures in research and therapy. The values of test-retest reliability range from about .56 to .87 and the internal consistencies range from Cronbach’s alpha = .62 to .95. Furthermore, the studies mentioned above examined the construct and criterion validities of the instruments and showed that they are also sufficiently valid.

### Plans to promote participant retention and complete follow-up {18b}

For motivation, the benefits resulting for the participant will be highlighted, e.g., comprehensive diagnostics, insights into VR or eye-tracking glasses as well as the expense allowance. Especially the AA needs to be strengthened; therefore, a bonus in the expense allowance will be promised for conscientious participation.

No further data will be collected from the participants if participation is discontinued.

### Data management {19}

A double coding of at least 30% of the data (questionnaires, mood ratings, processing of gaze data) is planned. Electronic data (e.g., heart rate and collected data during AA) will be transmitted without processing. The saliva samples will be analyzed once. As a security concept, the data will be stored locally with local backup.

### Confidentiality {27}

Personal information, study data, and the list of assignments between participant code and personal data will be stored separately and locked away. The assignment list will be destroyed 1 year after completion of the study, whereby the participant data will be anonymized. Until then, the data will only be processed pseudonymously under the participant code. During analyses, the saliva samples will be passed on to an external laboratory that is subject to data protection and privacy law. Furthermore, the saliva samples will only be labelled with participant code.

### Plans for collection, laboratory evaluation, and storage of biological specimens for genetic or molecular analysis in this trial/future use {33}

The saliva samples will be collected by the participants themselves during the AA, stored in the refrigerator and brought along for the testing appointment. These and the samples collected during the testing procedures will be stored at −20 °C locally until shipping to the laboratory. After analysis of the hormone concentrations in the external laboratory, the samples will be stored additional 6 months to enable control analyses for outliers. Subsequently, they will be destroyed, and thus, further storage or analysis will not be possible.

## Statistical methods

### Statistical methods for primary and secondary outcomes {20a}

The parameters of the objective stress reactions will include heart rate, gaze behavior, cortisol, and alpha-amylase. For the analysis of the subjective stress reactions, we will use the self-reported mood ratings of the participants. The saliva samples will be determined by an external laboratory to analyze cortisol and alpha-amylase responses. For statistical analysis, we will use the software IBM SPSS Statistics 28 (IBM Corp., Armonk, NY) and R [52]. After checking the normal distribution of the data, parametric or non-parametric tests will be applied accordingly. Possible differences in the responses of cortisol, alpha-amylase, heart rate, gaze behavior, and mood ratings between the groups (between-subject factors: TSST-VR vs. TSST-IV, HCE vs. NR) and over time (inter-subject factor) will be investigated by means of (repeated measures) analyses of variance ((rm)ANOVAs, parametric) or Mann-Whitney *U* tests / Friedman tests (non-parametric), and post hoc *t*-tests. Equivalence tests will also be used to investigate a comparable increase in the stress responses between the TSST conditions. For this purpose, mean or median values will be calculated from the variables for each group, depending on the distribution of the data and the test used. Area under the curve (AUC) values will also be calculated for analysis of changes in hormonal stress responses [53]. Possible associations between subjective sleep quality / quantity, mood, personality traits and hormonal awakening responses, diurnal profiles, and stress reactions will be analyzed by means of correlations and regression analyses. Again, parametric (e g., Pearson correlation) or non-parametric tests (Spearman or Kendall correlation) will be used depending on the normal distribution and scale level of the data. For multiple comparisons, the *p* values will be corrected according to the false discovery rate (FDR) [54]. Here, the primary outcome variables are corrected collectively across all parameters due to the underlying common hypothesis of a multimodal stress response to the TSST. The statistical significance level will be set to *α* = .05.

### Interim analyses {21b}

Interim analyses will not be necessary due to the preceding telephone screening (T0). The study will be terminated once 84 participants have been completely tested.

### Methods for additional analyses (e.g., subgroup analyses) {20b}

Subgroup analyses for age, sex, and school type are planned. The analyses of the relaxation reactions will be adjusted for the TSST condition if there will be differences in the stress responses between the TSST-IV and the TSST-VR.

### Methods in analysis to handle protocol non-adherence and any statistical methods to handle missing data {20c}

If data is missing, a distinction will be made between data missing at random and missing not at random by computing a Missing Completely At Random (MCAR) test. For data missing at random (e.g., contaminated saliva samples), multiple imputation and maximum likelihood estimation can be used [55]. Completely missing data (e.g., due to failure of technical components) will be excluded. The handling of missing data in questionnaires will depend on the recommendations in the respective manual. Outliers (e.g., due non-adherence) will also be analyzed and excluded if necessary.

### Plans to give access to the full protocol, participant-level data, and statistical code {31c}

In line with the Open Science concept, it is planned to publish the participant-level datasets in anonymous and aggregated form. Furthermore, the protocol and statistical code will be provided by the investigators upon request.

## Oversight and monitoring

### Composition of the coordinating center and trial steering committee {5d}

The study will be a single-center study. Responsible and involved persons will be staff members of the Clinic of Child and Adolescent Psychiatry, Psychosomatics, and Psychotherapy. The responsibility will exclusively lie with the study management. No other persons will be involved.

### Composition of the data monitoring committee, its role and reporting structure {21a}

No data monitoring committee is planned in this study. The reasons for this are, apart from the parallel interventions, the manageable planned sample size of *N* = 84 and the single-center design of the study. This also means that there are no competing interests.

### Adverse event reporting and harms {22}

Adverse events could occur in this study, particularly during AA and stress induction. Since the AA will be conducted via smartphones, technical errors cannot be excluded. Any problems that occur during the AA will be queried using the app itself (e.g., “Were there any technical problems with the app?”), and queried again at T2 and noted on a protocol. If the input will not work or the reminders of the app will be missing completely, the AA can be repeated. For this purpose, participants can contact the study management.

During stress induction in VR, VR sickness can occur despite precautions. Should a break be necessary, this will be noted in detail to document a shift in the schedule. Depending on the extent of the disturbance, the data will be excluded from the evaluation.

If the stress induction is too stressful and not bearable for the participant, the TSST will be interrupted. Experience shows that this is very unlikely. In general, the survey could be perceived as burdensome by children and adolescents. If this becomes too stressful for the participants, breaks can be taken or in extreme cases the investigation can be aborted. However, this will also lead to exclusion from the study. All adverse effects (e.g., high stress levels and VR sickness) are noted in detail on a protocol.

In summary, possible deviations from the planned procedure will always be collected and reported.

### Frequency and plans for auditing trial conduct {23}

No audit of the trial conduct is planned.

### Plans for communicating important protocol amendments to relevant parties (e.g., trial participants, ethical committees) {25}

Important protocol changes will be communicated to the parties concerned in advance (ethics committee) or upon implementation (study registry, journals).

### Dissemination plans {31a}

Participants can receive their results from the questionnaires and the intelligence test on request. These will be prepared in an understandable way. If interested, the results of the main questions of the study can also be reported back.

In general, the results of the study will be published in anonymous form. Publications are planned in peer-reviewed journals as soon as the data have been fully collected and analyzed.

## Discussion

To the best of our knowledge, this study will represent the first controlled-randomized study to investigate stress reactions caused by a virtual TSST compared to a real TSST in children and adolescents. By using a virtual TSST version, several research disadvantages can be overcome, e.g., reduced effort in stress induction and better control of potential confounding variables. Thus, stress research in the field of children and adolescents can be further enhanced. The lack of studies in this area is unfortunately very similar to other areas of application of VR in childhood and adolescence, as for example the review by Kothgassner [56] on VR exposure therapy for anxiety disorders in childhood and adolescence shows. An effective applicability can be expected, but the corresponding studies are still to be conducted. Another innovative aspect of this study will be the inclusion of gaze behavior as a stress parameter. A study by Herten and colleagues [57] provides initial evidence of its suitability as a stress parameter, showing that under psychosocial stress, social stimuli such as faces are avoided to a greater extent. The comparability of gaze behavior between the real and the virtual version will also be considered in connection with the acute stress reaction. The everyday stress level will be determined in advance in this study by performing an AA. This will provide a better insight into the interactions of everyday and acute stress reactions, possible dysregulations, and conceivable correlations with factors such as sleep, childhood adversities, or personality traits in childhood and adolescence [58]. In addition to the stress induction experiment, a relaxation exercise for stress reduction will be evaluated in comparison to an unguided relaxation. Due to the short duration of the application and the absence of a long-term training phase, this exercise represents a very low-threshold offer, which could be easily applied in everyday life as well as in clinical settings.

It is expected that participants will show an acute stress reaction on several levels (physiological, hormonal, subjective, behavioral) induced by the TSST-VR, which is approximately to the same extent as the acute stress reaction of participants in the TSST-IV. If this is the case, the more elaborate TSST-IV can be omitted in future studies and acute stress can only be induced with a virtual TSST. This would also provide evidence that VR is generally well suited for use in childhood and adolescence. An advantage of HCE as a relaxation exercise over NR is expected. If this becomes apparent, a very simple, low-threshold tool would be evaluated, with which an acute stress reaction can be better managed than with NR after a stressful situation. The data from the AA will provide information on the diurnal hormonal profile, as well as insights into sleep quality / quantity, stressful events, and used coping strategies. These data can then be considered in relation to each other and to the acute stress reaction, thus further contributing to the general pattern.

The following potential limitations or challenges may arise in the three main areas of the study: Regarding stress induction, not all parameters in both groups may respond to the induction with equal strength. Firstly, non-responders are part of everyday life in stress research [59], and secondly, a stress response is not always evident at all levels. Therefore, a multimodal psychometric and biological measurement is provided [58]. Based on this, more reliable statements about the stress response can be made. Possible influences of sex or age on the various stress parameters are addressed by the inclusion and exclusion criteria and randomization across groups. A potential influence of the VR environment itself on the stress level in children and adolescents has not yet been investigated. Factors such as VR sickness and presence could have an influence and will therefore be documented in this study. During the AA, no control of the collection of data (e.g., saliva samples and subjective data) will be feasible, but will be compensated as much as possible by the use of a medication event monitoring system, previous demonstration of saliva sample collection and app use, notifications via the app in clear time limits, and reward bonuses. Technical problems cannot be completely excluded, but in such cases it is possible to contact the study personnel in order to minimize data loss. During the relaxation exercise, there will be no recording of whether the breathing exercise is performed correctly in the HCE and whether the NR really does not include any breathing exercise. However, the presence of the experimenter is believed to have a positive effect on the correct execution according to the instructions.

In summary, this study aims to improve the understanding of the mental and physiological effects of acute and everyday stress and the applicability of a virtual stress paradigm to investigate acute stress and the stress-reducing effect of an HCE in children and adolescents. Findings of this study may facilitate future stress research in the field of children and adolescents and may lead to an expanding use of these laboratory stressors paradigms in clinical and population-based trials.

## Trial status

The recruitment of participants started in June 2021 and will be completed until January 2023. The protocol is in its third version from 05 September 2022.

## Data Availability

The datasets used and/or analyzed during the current study are available from the corresponding author on reasonable request.
